# A Research Agenda for Appearance Changes Due to Breast Cancer Treatment

**DOI:** 10.4137/bcbcr.s784

**Published:** 2008-06-16

**Authors:** Mugdha Dabeer, Michelle Cororve Fingeret, Fatima Merchant, Gregory P. Reece, Elisabeth K. Beahm, Mia K. Markey

**Affiliations:** 1 The University of Texas Department of Biomedical Engineering, Austin, TX; 2 Department of Behavioral Sciences, The University of Texas M. D. Anderson Cancer Center, Houston TX; 3 Department of Plastic Surgery, The University of Texas M. D. Anderson Cancer Center, Houston TX; 4 Soft Imaging, League City TX

**Keywords:** breast cancer, 3D imaging of breast, computer-assisted image analysis, quality of life

## Abstract

Breast cancer is one of the most prevalent forms of cancer in the US. It is estimated that more than 180,000 American women will be diagnosed with invasive breast cancer in 2008. Fortunately, the survival rate is relatively high and continually increasing due to improved detection techniques and treatment methods. However, maintaining quality of life is a factor often under emphasized for breast cancer survivors. Breast cancer treatments are invasive and can lead to deformation of the breast. Breast reconstruction is important for restoring the survivor’s appearance. However, more work is needed to develop technologies for quantifying surgical outcomes and understanding women’s perceptions of changes in their appearance. A method for objectively measuring breast anatomy is needed in order to help both the breast cancer survivors and their surgeons take expected changes to the survivor’s appearance into account when considering various treatment options. In the future, augmented reality tools could help surgeons reconstruct a survivor’s breasts to match her preferences as much as possible.

## Quantitative Assessment of Breast Aesthetics

The quality of life of a patient with breast cancer can be greatly enhanced by minimizing the adverse effects that the treatment of the disease may impart on physical appearance. Aesthetic outcome is an important endpoint of breast cancer treatment. Recently, we presented an extensive review of the literature on methodology for the quantification of breast aesthetics (Kim, Sbalchiero et al. 2008). In the next few paragraphs, we briefly summarize the state-of-the-art in assessment of breast aesthetics to provide context for the remainder of the commentary.

Qualitative, subjective scales have largely been utilized to assess aesthetic outcome. However, none of these scales has achieved widespread use, likely because they are generally vague with a low intra- and inter- observer agreement.

Other studies have made use of anthropometry, which is an approach to quantifying breast aesthetics using “linear measurements” between specific anatomical landmarks (fiducial points) on a patient. Common fiducial points utilized in breast analysis include the nipples, umbilicus (navel), and sternal notch, and humerus (arm). A few authors have made linear measurements with the intent of establishing standard values, but such reference values have not been widely adopted and comparison to outcomes are lacking. Relationships between linear measurements and subjective assessments are unclear. Since linear measurements aren’t routinely collected, studies require an additional intervention and it isn’t feasible to make a large number of measurements on each subject. If a particular measurement doesn’t prove valuable, one can’t retrospectively try a different one.

It is possible, however, to perform many of the same linear measurements on a photograph of the patient instead of on the patient directly. Recent studies have demonstrated the potential of photogrammetry in evaluating breast aesthetics (Kim, Reece et al. 2007). Moreover, by using digital/digitized photographs, it is possible to automate the process more fully by using image processing techniques to identify fiducial points (Udpa, Sampat et al. 2007). In addition to deceasing barriers to use, automatic identification of fiducial points will also make the measures of breast aesthetics more reliable by eliminating the need for human observers. Another avenue for future research is use color information, not just geometric relationships, to quantify scarring and other skin properties. We recently demonstrated that digital photography is an accurate alternative to using dedicated colorimetry equipment (Kim, Rodney et al. 2008).

However, two-dimensional (2D) linear measures, based on either direct clinical measurement (anthropometry) or photogrammetry, are inherently limited for documenting the three-dimensional (3D) volume and curvature of the breast. Thus, 3D surface imaging is needed to fully describe appearance changes due to breast cancer treatment.

## 3D Imaging

The application of 3D imaging is a relatively recent innovation as a tool in the assessment of breast appearance. This technology creates a digital 3D image of the body surface, which looks like a virtual sculpture of the subject. Several technologies, such as stereophotogrammetry, laser scanning, 3D digital photography, and light digitizers, are used to create 3D images that have advantages in the analysis of human physique (Honrado and Larrabee, 2004). 3D imaging is already in widespread use in analysis of craniofacial and facial surgery (Malata, Boot et al. 1994). 3D digital photography systems are capable of non-invasively and quickly capturing high-definition volumetric image data and constructing topographic surface maps of the breast that permit accurate evaluation and objective determination of differences in volume, surface area, shape, size, contour, and symmetry (Malata, Boot et al. 1994). A single 3D image yields more information regarding breast appearance than multiple conventional photographs on some elements of the breast appearance, such as volume, that are not available from two-dimensional images.

3D machines currently on the market for plastic surgery are stereophotogrammetry systems designed to project a random light pattern on the patient and capture her with precisely synchronized digital cameras set at various angles in an optimum configuration. The 3D surface geometry and surface texture of the patient are simultaneously acquired.

However, while equipment for capturing 3D surface images is available, there is a decided lack of validated software tools to enable those images to improve patient care. An active area of research is to develop algorithms that would aid the surgeon in quantifying and interpreting 3D data in a clinically relevant fashion (Gupta, Han et al. 2007). While visualization tools and quantitative measures are important steps, it is critical to recognize that simple image morphing programs that have no basis in biomechanics are of little value and may instead create new problems as they can give rise to unrealistic expectations. Ultimately, we seek tools not only to help doctors communicate visually with patients, but also to provide real-time, intra-operative guidance.

## Augmented Reality

Augmented reality enhances information associated with a real object. It does not simulate reality, but uses available information and technology to add context to the data. It change’s one perception of a real object by adding graphical effects and other types of tangible information, like sound, smell, and motion. While in many ways virtual reality is similar to augmented reality, there are distinct differences (JH, 2004). In both the cases, by superimposing, images, videos, or text onto real life, an experience can be heightened or even modified. However, virtual reality simulates unavailable data, whereas augmented reality modifies one perception of real data. The application of the technology for the analysis of outcomes in breast surgery could be extrapolated to enhance surgical planning and documentation, as well as teaching assistance. In the future, students can visualize on a computer interface both original and anticipated outcomes of a particular surgical intervention in comparison to the desired images of the breast—all overlaid on one another (Rohrich, Adams et al. 2007).

Intra-operatively, surgeons are generally forced to plan and assess their intervention by changing their field of vision by looking away from the operating site and comparing the reconstructed breast to a static graphic scan or representation. In the future, using instruments with augmented reality, the surgeon’s headgear would sense his/her line of sight. The instrument would then project the desired image on the surgeon’s goggles. Thus, augmented reality would allow them to maintain a fixed field of vision on the surgical site (JH, 2004). Going further, augmented reality might add audio commentary, location data, historical context, or other forms of content that can make a doctor’s experience of a surgery more meaningful.

However, applying augmented reality in the operating room is very difficult in practice. The technology must maintain a full view of the patient while superimposing virtual images on the operative site.

## Quality of Life and Patient Satisfaction

At the heart of our work is the desire to not only improve surgical techniques but to maximize patient’s quality of life and satisfaction with breast reconstruction outcomes. Augmented reality tools have the potential to be particularly valuable in helping surgeons reconstruct a survivor’s breast to match her stated preferences. This requires further understanding and consideration of a host of psychosocial variables that can influence patient-reported outcomes. Available research focusing on patient satisfaction in breast cancer patients can be substantially improved upon with more rigorous study designs that address the complex process of reconstructive treatment.

## Conclusion

The goal of quantitative breast assessment techniques is to develop software tools that would aid the surgeon in quantifying and interpreting appearance changes due to breast cancer treatment in a clinically relevant fashion. The use of 3D images is an important component since important breast aesthetic features such as volume and shape cannot be fully assessed from clinical photographs. Augmented reality could be implemented in routine surgical practice in the foreseeable future and complement and improve surgical procedures in many ways.
